# Effects of Dexmedetomidine on the Behavioral Outcomes in Streptozotocin‐Induced Alzheimer's Disease Rats

**DOI:** 10.1002/brb3.71196

**Published:** 2026-01-07

**Authors:** Mina Mohasel‐Roodi, Masoumeh Nozari, Ali Shamsara, Mohsen Basiri, Vida Mirzaie, Masoumeh Baghalishahi

**Affiliations:** ^1^ Department of Anatomy, Afzalipour School of Medicine Kerman University of Medical Sciences Kerman Iran; ^2^ Neuroscience Research Center, Institute of Neuropharmacology Kerman University of Medical Sciences Kerman Iran

**Keywords:** Alzheimer's disease, animal model, dexmedetomidine, streptozotocin

## Abstract

**Introduction:**

Alzheimer's disease (AD) is a progressive and prevalent neurodegenerative disorder characterized by progressive cognitive decline and memory impairment. Intracerebroventricular (ICV) administration of streptozotocin (STZ) in rodents recapitulates key features of sporadic AD, including brain insulin resistance and oxidative stress. Dexmedetomidine (Dex), a highly selective α2‐adrenergic receptor agonist, has demonstrated neuroprotective and anti‐inflammatory properties, suggesting its potential utility as a therapeutic approach for AD.

**Methods:**

Seventy adult male Wistar rats were randomly allocated to seven experimental groups: Control, Sham, STZ, Sham + Dex (25 µg/kg), and STZ + Dex (25, 50, 100 µg/kg). Cognitive performance and anxiety‐like behaviors were evaluated using the open‐field test (OFT), elevated plus maze (EPM), Y‐maze test, and Morris water maze (MWM).

**Results:**

In the Y‐maze, STZ‐treated rats exhibited significant reductions in spontaneous alternation behavior (*p* = 0.002), which were significantly reversed by Dex (25 µg/kg, *p* = 0.002). In the MWM, the STZ administration resulted in prolonged escape latencies and increased path lengths compared with Control animals (*p <* 0.05). Treatment with Dex (25 µg/kg) significantly improved spatial learning and memory retention (*p <* 0.05). No significant differences were observed in locomotor activity and anxiety‐related behaviors in the OFT or EPM.

**Conclusions:**

These findings indicate that Dex at 25 µg/kg attenuates STZ‐induced cognitive deficits, likely through neuroprotective and anti‐inflammatory mechanisms. The results highlight Dex as a promising candidate for AD therapy, though further research is required to elucidate its underlying molecular pathways. The study supports the potential repurposing of Dex for neurodegenerative disorders.

## Graphical abstract

Dexmedetomidine at 25 µg/kg effectively reverses working and spatial memory deficits in a streptozotocin (STZ)‐induced rat model of Alzheimer's disease, restoring Y‐maze alternation and Morris Water Maze performance. Higher doses show no clear benefit, highlighting Dex‐25 as a promising neuroprotective candidate.

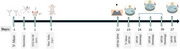

## 1 Introduction

Alzheimer's disease (AD) is one of the most common and debilitating age‐associated neurodegenerative disorders. Clinically, AD is characterized by a gradual decline in cognitive abilities, progressive memory impairment, and a spectrum of behavioral disturbances (Kim et al. 2024; Zhang et al. 2024). Recent estimates indicate that the global number of individuals across the AD continuum—including those with dementia, prodromal disease, and preclinical biomarker evidence—now exceeds 100 million, with approximately 32 million people currently living with AD dementia (Frisoni et al. 2025). Despite substantial advances in identifying genetic, molecular, and environmental risk factors, the etiology of AD remains highly multifactorial. Contemporary mechanistic research indicates that multiple interacting pathways collectively drive disease progression. Beyond the classical amyloid‐β cascade and tau hyperphosphorylation, chronic neuroinflammation and immune dysregulation have emerged as major contributors to neuronal injury, with peripheral immune cells infiltrating the brain and amplifying cytokine‐mediated neurotoxicity (Zhang et al. 2024). Vascular dysfunction—including blood–brain barrier disruption, impaired cerebral perfusion, and endothelial injury—further exacerbates amyloid and tau pathology and reduces the brain's resilience to neurodegeneration (Mekala and Qiu 2025; Svenningsson et al. 2024). In addition, disturbances in lipid metabolism and mitochondrial dysfunction have been identified as interconnected processes contributing to disease heterogeneity (Cao et al. 2024; Kashif et al. 2023).

*α*_2_‐Adrenergic receptors (α_2_‐ARs) are G‐protein–coupled receptors widely expressed in the locus coeruleus, hippocampus, and prefrontal cortex, where they regulate noradrenergic transmission through presynaptic inhibition of norepinephrine release. Activation of α_2_‐ARs reduces cyclic adenosine monophosphate (cAMP) signaling, decreases neuronal excitability, and modulates downstream signaling pathways involved in inflammation and oxidative stress (Gannon et al. 2015).

Noradrenergic dysfunction is increasingly recognized as a critical contributor to AD pathophysiology, largely due to early degeneration of locus coeruleus neurons, reduced cortical norepinephrine levels, and impaired microglial regulation—changes that promote chronic neuroinflammation, synaptic vulnerability, and cognitive decline (Beardmore et al. 2021; Gannon et al. 2015). Within this mechanistic context, dexmedetomidine (Dex), a highly selective α_2_‐ARs agonist, has gained increasing attention for its ability to attenuate neuroinflammation, limit oxidative stress, and preserve synaptic integrity in preclinical models of neurodegeneration (He et al. 2023; Hu et al. 2022). Moreover, Dex has been shown to counteract β‐amyloid–induced neurotoxicity and to enhance brain‐derived neurotrophic factor expression (BDNF) (Deng et al. 2024; Yu et al. 2022). Experimental studies further report improved cognitive performance and neuronal protection in β‐amyloid–challenged mouse models following Dex treatment (Sun et al. 2020).

Dex is widely used in anesthetic practice because of its sedative and sympatholytic properties, and clinical evidence indicates that it reduces both the incidence and severity of postoperative delirium compared with other sedatives (Flükiger et al. 2018). Collectively, these mechanisms support Dex as a potential therapeutic strategy for ameliorating cognitive dysfunction associated with AD, either as a direct intervention or as an anesthetic adjunct in affected patients.

The present study investigated the effects of Dex on behavioral abnormalities in a streptozotocin (STZ)‐induced rat model of AD, which is widely regarded as a translational model of sporadic AD pathology. STZ, originally identified for its diabetogenic effects, has been utilized in neuroscience research to mimic AD‐like phenotypes. Intracerebroventricular (ICV) STZ administration disrupts cerebral insulin signaling, leading to neuronal dysfunction, oxidative stress, and cognitive impairment—hallmarks of sporadic AD (Grieb 2016). Although Dex has shown beneficial effects in other AD‐related models, its evaluation in the STZ paradigm may provide clinically relevant insight, given that sporadic AD constitutes the majority of cases in humans.

## 2 Materials and Methods

### 2.1 Experimental Animals

Adult male Wistar rats (2‐3 months old, weighing 200–250 g) were obtained from the Animal Care Center of Kerman University of Medical Sciences (Kerman, Iran). Animals were housed under standard laboratory conditions (temperature 21 ± 2°C, relative humidity 60%–65%) with a 12:12 h light–dark cycle and ad libitum access to food and water.

The seventy rats were randomly assigned to seven experimental groups (*n* = 10): (1) Control, which received no surgery or treatment; (2) Sham, which received bilateral intracerebroventricular (ICV) injections of artificial cerebrospinal fluid (ACSF; 147 mM NaCl, 2.9 mM KCl, 1.6 mM MgCl_2_, 1.7 mM CaCl_2_, and 2.2 mM dextrose) through cannulas implanted into the lateral ventricles (coordinates from Paxinos and Watson: AP: 0.8 mm; ML: 1.5 mm; DV: 3.6 mm), at the same volume and time points as STZ administration, along with intraperitoneal (i.p.) injections of distilled water equivalent to the Dex vehicle volume;

(3) STZ, which received bilateral ICV injections of streptozotocin (3 mg/kg; 5 µL; Sigma‐Aldrich, Darmstadt, Germany) on days 1 and 3. The STZ animals did not receive an additional Dex vehicle because the vehicle consisted solely of sterile normal saline, which had already been administered during the ICV procedure in all groups and does not exert behavioral or neurobiological effects; 4) Sham + Dex 25, which received ACSF as described above and Dex (25 µg/kg; 0.005%, Hospira, USA) administered intraperitoneally after each ACSF infusion; and (5–7) STZ + Dex 25, 50, and 100, which received STZ in combination with Dex at doses of 25, 50, or 100 µg/kg, respectively.

(4) Sham + Dex 25, which received ACSF as described above and Dex (25 µg/kg; 0.005%, Hospira, USA) administered intraperitoneally after each ACSF infusion; and (5–7) STZ + Dex 25, 50, and 100, which received STZ in combination with Dex at doses of 25, 50, or 100 µg/kg, respectively.

**FIGURE 1:**
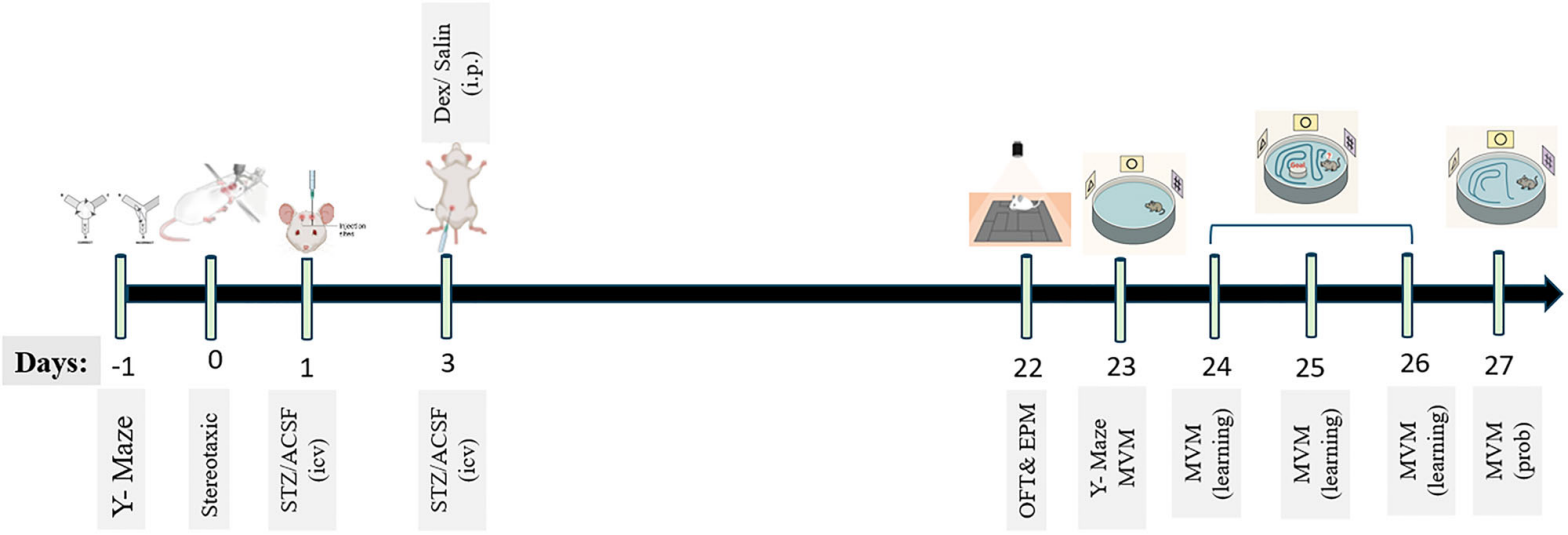
Experimental timeline of the study: ACSF, artificial cerebrospinal fluid; ICV, intracerebroventricular; i.p., intraperitoneal; Dex, dexmedetomidine; STZ, streptozotocin; OFT, open‐field test; EPM, elevated plus maze; MWM, Morris water maze.

A Sham + Dex (25 µg/kg) group was included to Control for potential behavioral effects of Dex in non‐STZ‐treated animals. The 25 µg/kg dose was selected based on previous studies demonstrating robust neuroprotective efficacy without inducing sedative confounds. In contrast, higher doses (50–100 µg/kg) were not administered to Sham animals to minimize animal use and avoid potential behavioral suppression. Dex was delivered via the intraperitoneal route, which provides reliable systemic absorption and minimizes stress associated with repeated intravenous administration. A schematic overview of the experimental design and timeline is presented in Figure 1.

Only male rats were used to minimize variability related to sex hormones, as estrous cycle–dependent fluctuations in locomotor activity, anxiety‐like behavior, and spatial memory performance may introduce additional confounds in behavioral studies (Beery and Zucker 2011; Prendergast et al. 2014). Moreover, sex‐specific differences in susceptibility to STZ‐induced cognitive impairment have been previously reported (Bao et al. 2017).

All animal care procedures and behavioral experiments were conducted in accordance with the ethical guidelines of the Kerman University of Medical Sciences Animal Ethics Committee (Ethics code: 402000357). Behavioral assessments were performed between 8:00 a.m. and 2:00 p.m.

### 2.2 Behavioral Tests

#### 2.2.1 Open‐Field Test (OFT)

The OFT was used to assess spontaneous locomotor activity, exploratory behavior, and repetitive movements. Each rat was individually placed in a transparent Plexiglas arena (90 × 90 × 30 cm), and its activity was recorded using an automated video‐tracking system (Borj Sanat, Iran). The arena floor was divided into 16 equal squares, with the central four squares defined as the center zone. Locomotor activity was quantified as total distance traveled, while the time spent in the peripheral versus central zones was considered an indirect index of anxiety‐like behavior. Given its limited specificity for anxiety assessment, OFT outcomes were interpreted as complementary to the elevated plus maze (EPM) (Ennaceur 2014; Prut and Belzung 2003).

An observer blinded to group allocation also manually scored vertical exploratory activity (rearing) and stereotyped behaviors, including grooming movements such as forepaw or mouth rubbing and head cleaning (Parvan et al. 2024).

#### 2.2.2 Elevated Plus Maze (EPM) Test

Anxiety is a common comorbidity in individuals with Alzheimer's disease. The elevated plus‐maze (EPM) is one of the most established behavioral assays to examine the balance between exploration and fear in rodents, relying on their innate aversion to open spaces. The apparatus consisted of two open arms and two enclosed arms (30 × 5 cm each), with the closed arms surrounded by 15‐cm‐high walls. A central platform (5 × 5 cm) connected the arms, and the maze was elevated 50 cm above the floor. Each rat was placed in the central square facing an open arm and allowed to explore freely for 5 min. The time spent in each arm and the number of arm entries were automatically recorded using a video‐tracking system (Borj Sanat, Iran) (Saeedi Goraghani et al. 2019).

#### 2.2.3 Y‐Maze Test

The Y‐maze task was employed to evaluate spatial working memory. Testing was conducted at two time points: one day before stereotaxic surgery (baseline) and 22 days after the final STZ/ACSF injection. The maze consisted of three identical arms (30 × 20 × 10 cm). Each rat was placed at the end of one arm and allowed to explore the maze for 8 min. Sessions were video‐recorded, and spontaneous alternation behavior—defined as consecutive entries into three different arms without repetition—was calculated as a percentage (Sahraei et al. 2022):

$$\begin{array}{ccc} & & \%\mathrm{Alternation}\;\mathrm{Behavior}\\ & & =\frac{\mathrm{Entries}\;\mathrm{into}\;\mathrm{three}\;\mathrm{different}\;\mathrm{arms}\;\mathrm{consecutively}\;}{\mathrm{Total}\;\mathrm{number}\;\mathrm{of}\;\mathrm{arm}\;\mathrm{entries}\;-\;2}\;\times \;100 \end{array}$$

#### 2.2.4 Morris Water Maze (MWM) Test

In this task, spatial learning and memory were assessed using a circular water maze consisting of a pool (150 cm diameter, 60 cm height) and a hidden escape platform (12 cm diameter). The water temperature was maintained at 25 ± 2°C. Extra‐maze visual cues were placed around the testing room. A camera positioned above the center of the pool recorded each trial, and animal performance was analyzed using an automated tracking system (Borj Sanat, Iran). To reduce stress, rats underwent a habituation session one day before training, during which they swam freely for 60 s without the platform. The experiment included two phases: acquisition (training) and probe (recall). During the training phase, each rat performed four trials per day across three consecutive days (each day defined as a block). For each trial, the animal was released into the pool from one of four quadrants and given 60 s to locate the hidden platform. If the rat failed to find it within the allotted time, it was gently guided to the platform. After every trial, the rat was returned to its cage and placed on a warming pad for 30 s to recover. On the fourth day, the probe test was conducted after the platform had been removed. Rats were released from the quadrant opposite the target location and allowed to swim freely for 60 s. The primary outcomes measured were the duration of time spent in the target quadrant and the percentage of distance traveled within the platform area (Aghaei et al. 2017; Salmani et al. 2022).

### 2.3 Statistical Analysis

To verify the data distribution and variance assumptions, the Shapiro–Wilk test was applied for normality, and the Brown–Forsythe test for homogeneity of variance. For analysis of the acquisition phase in the MWM and Y‐maze performance, a repeated‐measures two‐way ANOVA was conducted. Within‐group differences in the Y‐maze were further examined using paired‐samples t‐tests. Depending on whether the variance assumptions were met, one‐way ANOVA or Welch's ANOVA was applied to other behavioral measures. Post hoc comparisons were carried out using either Tukey's or Dunnett's test. Statistical analyses were performed with SPSS software (version 25.0, SPSS Inc., USA), while line graphs were generated using GraphPad Prism 9 (GraphPad Software Inc., San Diego, CA, USA). Data are presented as mean ± standard error of the mean (SEM), and statistical significance was defined at a threshold of *p* < 0.05.

## 3 Results

### 3.1 OFT

**FIGURE 2:**
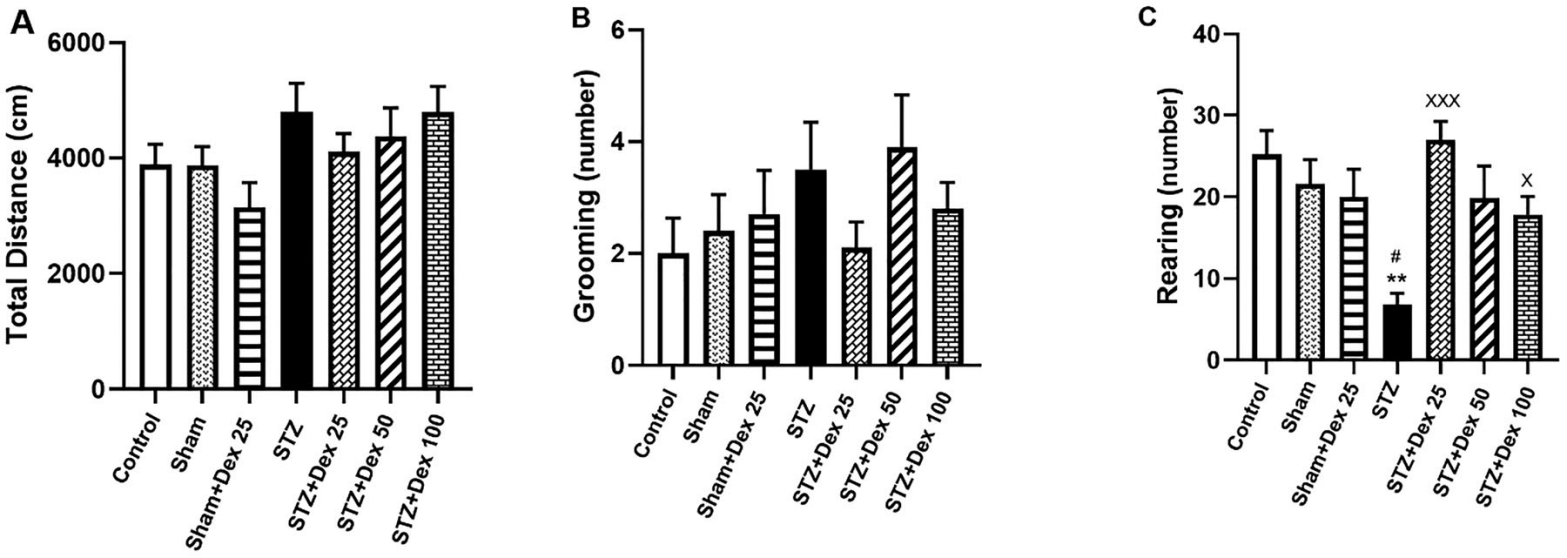
Effects of STZ and Dex on locomotor and exploratory behaviors in the open field test (OFT); (A) Total distance traveled and (B) grooming behavior showed no significant differences among experimental groups (*p* > 0.05). (C) Rearing behavior was significantly reduced in the STZ group compared with the Control and Sham groups and was significantly restored following Dex treatment at 25 µg/kg and 100 µg/kg. Data are presented as mean ± SEM (*n* = 10 per group). Statistical analysis was performed using one‐way ANOVA for panels A and B and Welch's ANOVA for panel C. **p < 0.01 vs. Control; #*p* < 0.05 vs. Sham group; × *p* < 0.05, ×××*p* < 0.001 vs. STZ group.

In the open field test, all variables except rearing behavior met the assumptions of normality and homogeneity of variance; therefore, one‐way ANOVA was applied for statistical comparisons. No significant effects of STZ or Dex treatment were observed on total distance traveled (Figure 2A), time spent in the center or peripheral zones (data are not shown), or grooming behavior (Figure 2B) (*p* > 0.05 for all comparisons).

Rearing data were normally distributed but exhibited heterogeneity of variance; therefore, Welch's ANOVA was employed. A significant group effect was detected for rearing behavior (W (6, 27.60) = 11.77, *p* < 0.0001). Rats in the STZ group displayed a marked reduction in rearing frequency compared with both the Control (*p* = 0.0017) and sham (*p* = 0.011) groups. Administration of Dex significantly restored rearing behavior in STZ‐treated animals, with significant improvements observed in the STZ + Dex 25 µg/kg (*p* = 0.0001) and STZ + Dex 100 µg/kg groups (*p* = 0.016) relative to the STZ group (Figure 2C).

### 3.2 Elevated Plus Maze (EPM)

**FIGURE 3:**
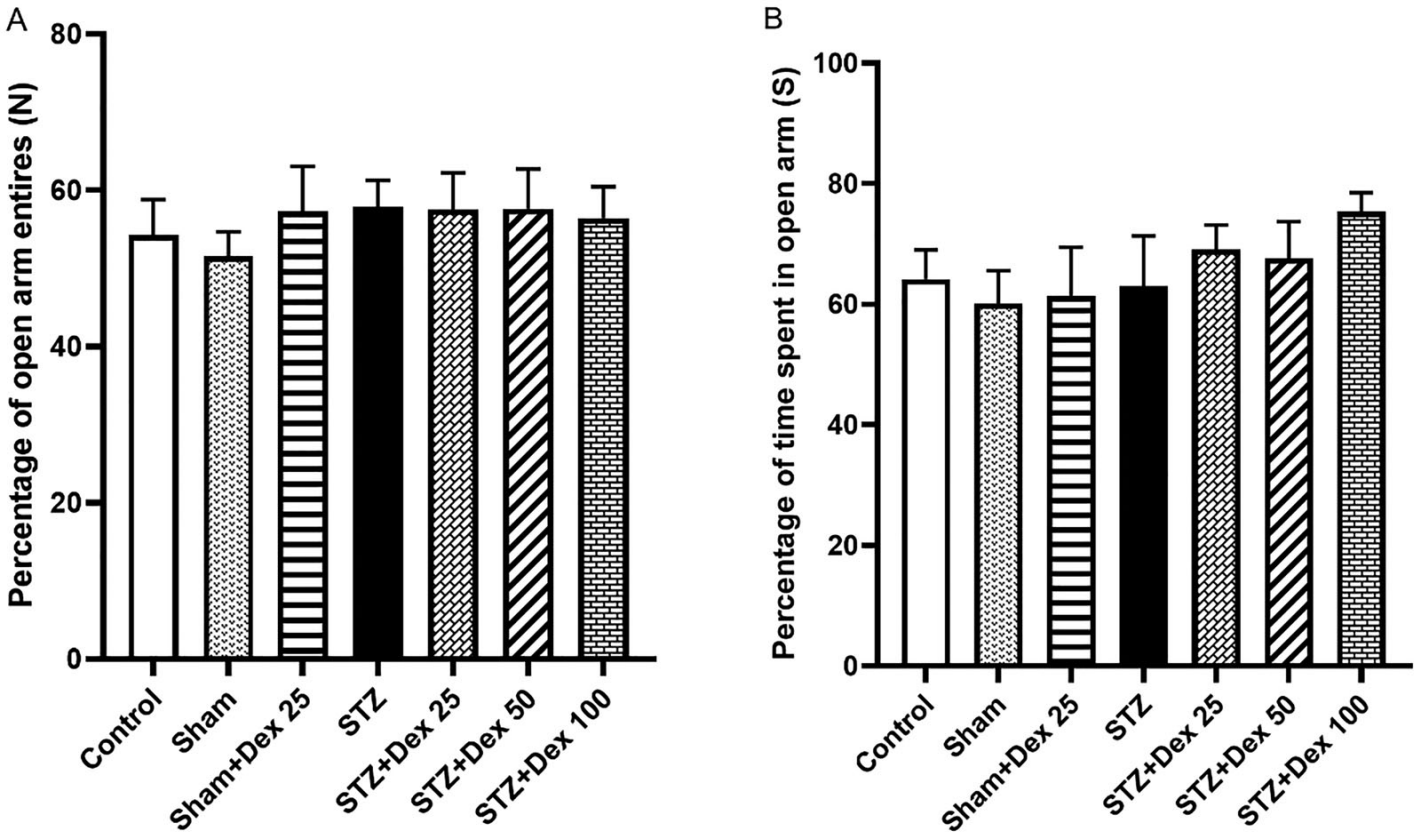
Effects of STZ and Dex on anxiety‐like behaviors in the elevated plus maze (EPM). (A) Percentage of entries into the open arms and (B) time spent in the open arms did not differ significantly among groups, indicating that neither STZ administration nor Dex treatment significantly affected anxiety‐related behavior. Data are expressed as mean ± SEM (*n* = 10 per group). Statistical analysis was performed using one‐way ANOVA.

To evaluate anxiety‐like phenotypes, the percentage of entry into open arms and time spent in open arms were analyzed. All EPM variables were normally distributed and showed homogeneous variances. One‐way ANOVA revealed no significant differences among experimental groups for either parameter (*p* > 0.05), indicating that neither STZ administration nor Dex treatment significantly altered anxiety‐related behaviors in this paradigm (Figure 3).

### 3.3 Y‐Maze Test

Y‐maze performance was analyzed using a two‐way repeated‐measures ANOVA, with time (pre‐ vs. post‐treatment) as the within‐subject factor and treatment group as the between‐subject factor. The analysis revealed a significant main effect of time [F(1,63) = 11.90, *p* = 0.001], indicating overall changes in performance across the testing sessions. A significant main effect of group was also observed [F(6,63) = 3.10, *p* = 0.007], suggesting differential effects of treatment conditions on working memory performance. Importantly, a significant group × time interaction was detected [F (6,63) = 3.39, *p* = 0.006], demonstrating that the magnitude of change from baseline differed among groups.

**FIGURE 4:**
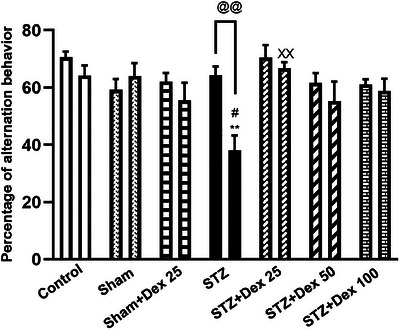
Effects of STZ and Dex on spontaneous alternation behavior in the Y‐maze test. STZ administration induced a significant reduction in spontaneous alternation behavior compared with baseline and with the Control and Sham groups, indicating impaired spatial working memory. Treatment with Dex at 25 µg/kg significantly reversed the STZ‐induced deficit, restoring alternation performance. Data are expressed as mean ± SEM (*n* = 10 per group). Statistical analysis was performed using two‐way repeated measures ANOVA followed by appropriate post hoc tests. **p < 0.01 vs. Control; #*p* < 0.05 vs. Sham group; ××*p* < 0.01 vs. STZ group; @@ *p* < 0.01 pre‐ vs. post‐treatment.

Post hoc analyses and paired‐samples t‐tests revealed that STZ administration resulted in a significant reduction in spontaneous alternation behavior (t (9) = 4.43, *p* = 0.002, pre‐ vs. post‐treatment), confirming the induction of spatial working memory impairment. Moreover, alternation performance in the STZ group was significantly lower than that observed in the Control and Sham groups (*p* < 0.01). Treatment with Dex at 25 µg/kg significantly reversed the STZ‐induced deficit, restoring alternation behavior relative to the STZ group (*p* = 0.002) (Figure 4).

These results indicate that STZ induces robust working memory impairment and that Dex confers a **protective cognitive effect**, particularly at the 25 µg/kg dose.

### 3.4 MWM

**FIGURE 5:**
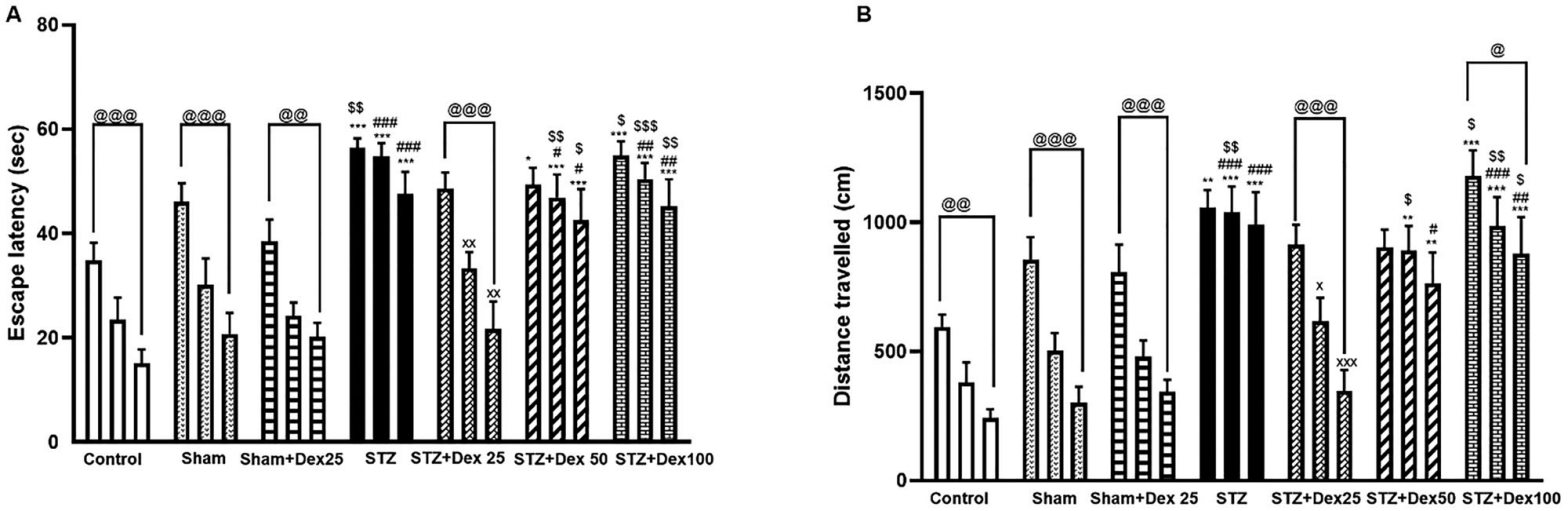
Effects of STZ and Dex on spatial learning during the acquisition phase of the Morris Water Maze (MWM) test. (A) Escape latency and (B) traveled distance across three training blocks. STZ‐treated rats exhibited significantly prolonged escape latencies and increased traveled distances compared with the Control and Sham groups, indicating impaired spatial learning. Treatment with Dex at 25 µg/kg significantly improved learning performance, as reflected by reduced escape latency and traveled distance. Data are expressed as mean ± SEM (*n* = 10 per group). Statistical analysis was performed using repeated‐measures two‐way ANOVA followed by post hoc comparisons. *p < 0.05, **p < 0.01, ***p < 0.01 vs. Control; #*p <* 0.05. ##*p <* 0.01, ###*p <* 0.001 vs. Sham group; ×*p <* 0.05, ××*p <* 0.01, ×××*p <* 0.001 vs. STZ group; @ *p <* 0.05, @@ *p <* 0.01; $*p <* 0.05, $$*p <* 0.01, $$$*p <* 0.01 vs. Sham + Dex 25, @@@ *p <* 0.001 vs. first block.

To evaluate the performance of spatial learning and memory, rats underwent an MWM task. During the acquisition phase, escape latency and traveled distance were analyzed across three training blocks using two‐way repeated‐measures ANOVA. The STZ group exhibited significantly prolonged escape latencies and greater traveled distances compared with the Control group (escape latency: blocks 1–3, *p <* 0.001; traveled distance: block 1, *p =* 0.004; blocks 2–3, *p <* 0.001) and the Sham group (escape latency: blocks 2–3, *p =* 0.002; traveled distance: block 2, *p =* 0.001; block 3, *p <* 0.001), indicating impaired spatial learning (Figures 5A, B).

Treatment with Dex at a dose of 25 µg/kg significantly ameliorated these learning deficits, as evidenced by reduced escape latencies (block 2, *p =* 0.0009; block 3, *p <* 0.01) and shorter traveled distances (block 2, *p =* 0.02; block 3, *p <* 0.001) compared with the untreated STZ group (Figures 5A, B).

Across the Control, Sham, Sham + Dex 25, and STZ + Dex 25 groups, a significant reduction in escape latency and traveled distance was observed in the third training block relative to the first block (*p <* 0.05), confirming effective learning across repeated trials (escape latency: Figure 5A, *p* < 0.05; traveled distance: Figure 5B, *p* < 0.05].

**FIGURE 6:**
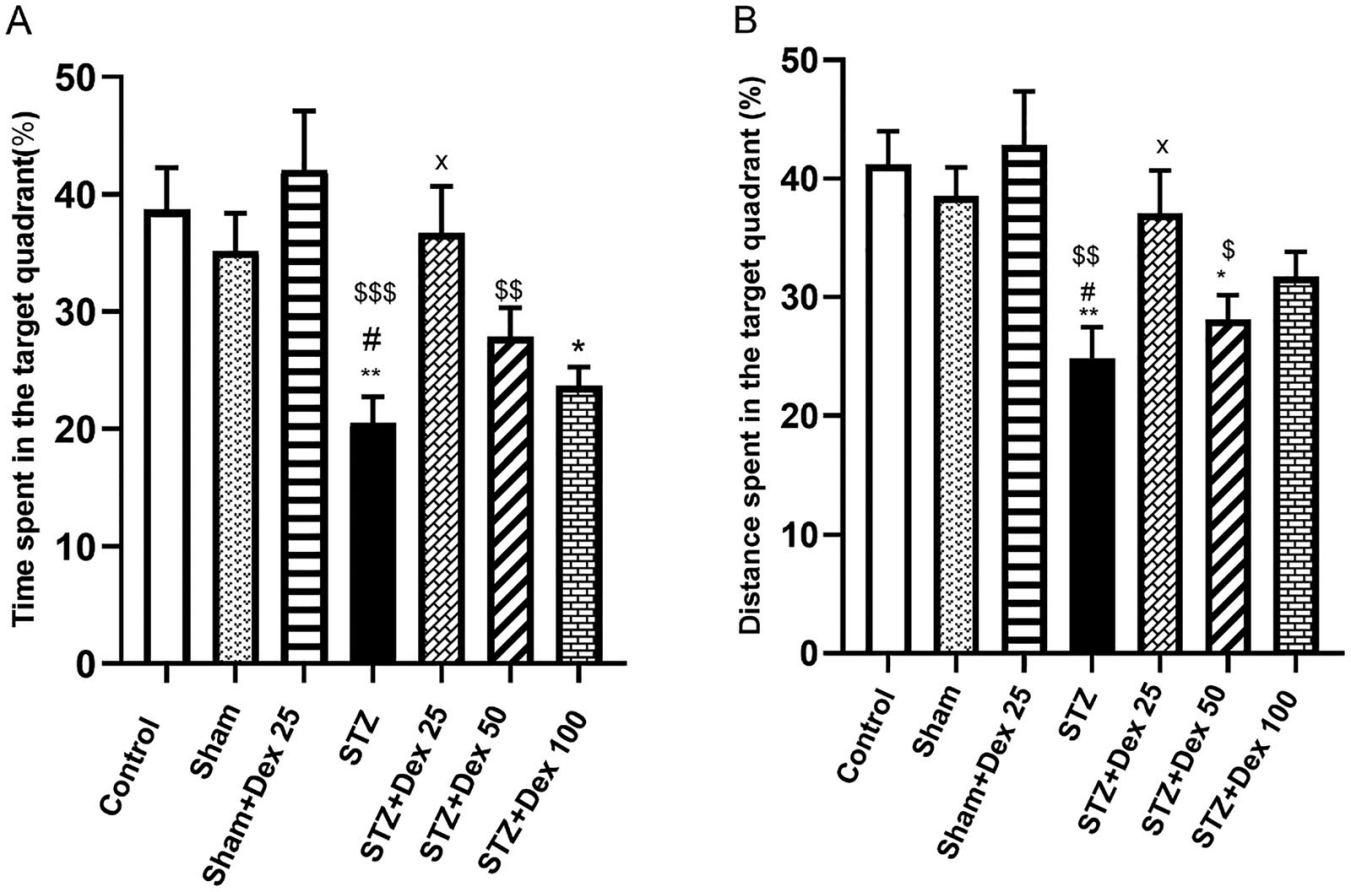
Effects of STZ and Dex on spatial memory retention in the probe trial of the Morris Water Maze (MWM) test. (A) Time spent and (B) distance traveled in the target quadrant following platform removal. STZ‐treated rats showed significantly reduced time and distance in the target quadrant, indicating impaired memory retention. In contrast, the STZ + Dex 25 µg/kg group exhibited significantly increased target‐quadrant exploration, reflecting improved spatial memory. Data are expressed as mean ± SEM (*n* = 10 per group). Statistical analysis was conducted using one‐way ANOVA followed by post hoc tests. *p < 0.05, **p < 0.01 vs. Control; #*p <* 0.05 vs. Sham group; ×*p <* 0.05 vs. STZ group.

In the probe trial, removal of the platform revealed that STZ‐treated rats spent significantly less time (*p <* 0.01 vs. Control) and traveled a shorter distance (*p <* 0.01 vs. Control) in the target quadrant, consistent with impaired memory retention. In contrast, rats in the STZ + Dex 25 µg/kg group spent significantly more time (*p <* 0.01) and traveled a greater distance (*p =* 0.03) in the platform quadrant compared with the STZ group, indicating improved spatial memory retention (Figures 6A, B). Collectively, these findings demonstrate that Dex at 25 µg/kg effectively reverses STZ‐induced deficits in both spatial learning and memory.

## 4 Discussion

The current research explored the potential therapeutic effects of Dex, a highly selective α_2_‐ARs agonist, on cognitive and behavioral impairments in an STZ‐induced rat model of AD. The findings demonstrated that Dex administration at a dose of 25 µg/kg significantly improved spatial learning and memory deficits, as assessed by the MWM and Y‐maze tasks. In contrast, anxiety‐like behaviors using the OFT and EPM were not significantly altered by either STZ administration or Dex treatment.

The observed cognitive benefits of Dex are consistent with previous studies reporting its capacity to modulate neuroinflammation, oxidative stress, and apoptotic signaling through α2‐adrenergic mechanisms—processes that are central to AD pathophysiology (Gannon et al. 2015; He et al. 2023; Hu et al. 2022). Improved performance in spatial memory tasks suggests that Dex may contribute to the preservation of hippocampal function and synaptic plasticity. The reversal of STZ‐induced deficits aligns with evidence indicating that Dex enhances BDNF expression, supports neurogenesis, and partially restores disrupted insulin signaling in experimental AD models (Arslan et al. 2022; Wang et al. 2018; Yonamine et al. 2023). Nevertheless, these mechanistic interpretations remain speculative, as the present study did not include molecular, biochemical, or histopathological analysis.

Although anxiety‐like behaviors were not prominently affected in the ICV STZ model used here, previous studies have reported considerable variability in affective outcomes following STZ administration. This inconsistency likely reflects model heterogeneity rather than a lack of pharmacological efficacy. Indeed, while some studies have observed altered anxiety‐related behaviors in younger animals, others using aged or behaviorally experienced rats have reported minimal changes in EPM performance (Gáspár et al. 2022; Rageh et al. 2012). Accordingly, the absence of anxiolytic‐like effects of Dex in the present study may reflect limited expression of anxiety phenotypes rather than true inefficacy. Future investigations incorporating stress‐enhanced or affect‐focused paradigms may better clarify this aspect.

Notably, the most robust neurocognitive benefits were observed at the 25 µg/kg dose of Dex. This finding is consistent with reports from other neurological models demonstrating optimal neuroprotection at lower doses, while higher doses may introduce sedative or hypomotor effects that confound behavioral assessment (Li et al. 2020; Ning et al. 2017; Xie et al. 2021). Thus, dose selection appears critical when evaluating Dex for cognitive outcomes.

Given its widespread clinical use and established short‐term safety profile, Dex represents an attractive candidate for repurposing as a drug in neurodegenerative conditions. Preclinical studies have consistently demonstrated that Dex attenuates neuroinflammation, oxidative stress, and synaptic dysfunction at clinically relevant concentrations, without major systemic toxicity (Deng et al. 2024; Yu et al. 2022). Small‐scale human studies have also explored Dex for the prevention of postoperative delirium and sedation in elderly or neurologically vulnerable patients, further supporting its tolerability when appropriately titrated (Devlin et al. 2018; Zhong et al. 2025). However, the potential risks associated with chronic α_2_‐adrenergic receptor stimulation—such as hypotension, bradycardia, and alterations in arousal states—must be carefully considered, particularly in elderly individuals or cognitively vulnerable populations (Weerink et al. 2017). Furthermore, the absence of long‐term safety data in the context of neurodegenerative disorders, along with potential species‐specific differences in central adrenergic signaling, necessitates a cautious interpretation of these findings. Accordingly, translating these promising preclinical results into effective therapeutic strategies will require well‐designed clinical trials that systematically evaluate optimal dosing regimens, treatment duration, and clinically relevant outcome measures in patients with Alzheimer's disease or other dementias.

## 5 Conclusion

This study demonstrates that Dex at 25 µg/kg effectively attenuated STZ‐induced impairments in spatial and working memory in a rat model of AD. No significant alterations were detected in anxiety‐like behaviors, which reflect the limited capacity of the ICV‐STZ model to elicit robust anxiety‐related phenotypes, rather than an absence of Dex efficacy in this behavioral domain. While the cognitive improvements observed suggest a potential neuroprotective role, the underlying mechanisms remain largely unresolved, as molecular and histological analyses were not performed. Collectively, these findings support Dex as a promising candidate for ameliorating AD‐associated cognitive decline; however, its mechanistic basis and long‐term safety profile must be clarified in future studies incorporating comprehensive neurobiological assessments.

## Author Contributions

**Mina Mohasel‐Roodi**: data curation, investigation, methodology. **Masoumeh Nozari**: conceptualization, supervision, writing – review and editing, project administration, funding acquisition. **Ali Shamsara**: writing – review and editing, supervision, methodology, **Mohsen Basiri**: writing – review. **Vida Mirzaie**: methodology. **Masoumeh Baghalishahi**: methodology.

## Funding

This research work was supported by the Kerman University of Medical Sciences, Kerman, Iran [grant number 402000357].

## Conflicts of Interest

The authors declare no conflicts of interest.

## Data Availability

The data that support the findings of this study are available from the corresponding author upon reasonable request.
